# Circulating Tumor DNA in Ovarian Cancer: Emerging Roles in Early Detection, Risk Stratification, and Disease Monitoring

**DOI:** 10.3390/cancers18081312

**Published:** 2026-04-21

**Authors:** Ludovica Pepe, Valeria Zuccalà, Walter Giuseppe Giordano, Giuseppe Giuffrè, Maurizio Martini, Vincenzo Cianci, Cristina Mondello, Massimiliano Berretta, Stefano Cianci, Vincenzo Fiorentino, Antonio Ieni

**Affiliations:** 1 Anatomic Pathology Unit, Department of Human Pathology in Adult and Developmental Age “Gaetano Barresi”, University of Messina, 98125 Messina, Italy; ludovica.pepe@studenti.unime.it (L.P.); valeria.zuccala@unime.it (V.Z.); giuseppe.giuffre@unime.it (G.G.); maurizio.martini@unime.it (M.M.); 2 PhD Program in Translational Molecular Medicine and Surgery, Department of Biomedical, Dental, Morphological and Functional Imaging Sciences, University of Messina, 98125 Messina, Italy; walter.giordano@studenti.unime.it; 3 Section of Legal Medicine, Department of Biomedical, Dental, Morphological and Functional Imaging Sciences, University of Messina, 98125 Messina, Italy; vincenzo.cianci@studenti.unime.it (V.C.); cristina.mondello@unime.it (C.M.); 4 Division of Medical Oncology, Department of Clinical and Experimental Medicine, University of Messina, 98125 Messina, Italy; massimiliano.berretta@unime.it; 5 Unit of Gynecology and Obstetrics, Department of Human Pathology in Adult and Developmental Age “Gaetano Barresi”, University of Messina, 98125 Messina, Italy; stefano.cianci@unime.it

**Keywords:** ovarian cancer, liquid biopsy, circulating tumor DNA, ctDNA methylation, CA-125, adnexal masses, early detection, molecular residual disease, multi-omic biomarkers

## Abstract

Early diagnosis of ovarian cancer remains one of the most important unmet needs in gynecologic oncology because survival is strongly stage-dependent and most patients still present with disseminated disease. Conventional non-invasive tools, particularly CA-125, transvaginal ultrasound, and composite triage algorithms, remain clinically useful but are limited by suboptimal sensitivity for stage I disease and by reduced specificity in premenopausal women and in benign inflammatory or endometriosis-associated conditions. Circulating tumor DNA (ctDNA) has therefore emerged as a candidate biomarker capable of extending liquid biopsy beyond conventional serology. In ovarian cancer, however, ctDNA implementation is constrained by low tumor shedding in early-stage disease, marked biologic heterogeneity across histotypes, clonal hematopoiesis-related background noise, and major pre-analytical and analytical sources of variability. This narrative review, informed by structured searches of PubMed, Scopus, and Web of Science, examines the evolving evidence for ctDNA mutations, methylation-based assays, multi-omic platforms, and machine-learning models across three distinct clinical contexts: population screening, preoperative triage of adnexal masses, and post-treatment assessment of molecular residual disease. We also discuss positive predictive value, false-positive harms, health-economic implications, standardization initiatives, and ongoing prospective studies. Overall, current evidence suggests that the most plausible near-term role for liquid biopsy in ovarian cancer is not as a universal stand-alone screening test, but as an integrated component of risk stratification and disease-monitoring frameworks that combine molecular signals with clinicopathologic and imaging data.

## Simple Summary

Liquid-biopsy strategies in ovarian cancer are advancing rapidly, but their potential differs markedly according to the clinical setting in which they are used. In population screening, the main obstacle remains stage I disease, where tumor shedding is minimal and extremely high specificity is required to avoid large numbers of false-positive results. By contrast, in symptomatic women with adnexal masses and in post-treatment surveillance, cfDNA methylation assays, multi-analyte models, and tumor-guided ctDNA monitoring appear more clinically plausible in the near term. This review critically examines mutation-based ctDNA assays, methylation classifiers, and multi-omic platforms, while emphasizing the need to distinguish proof-of-concept studies from true clinical utility, to control for pre-analytical variation and clonal hematopoiesis, and to validate assays prospectively within clearly defined ovarian-cancer pathways.

## 1. Introduction

As the deadliest gynecological malignancy and one of the most prevalent cancers affecting women worldwide, ovarian cancer remains a major global health challenge [1,2]. Recent GLOBOCAN 2022 estimates indicate approximately 324,398 new diagnoses and 206,839 disease-related deaths worldwide each year [2].

The clinical presentation of epithelial ovarian cancer (EOC) is characterized by insidious onset and nonspecific symptomatology. Abdominal bloating, early satiety, pelvic pain, and gastrointestinal discomfort are common complaints that may initially be misattributed to benign physiological or digestive conditions. More than 58% of patients report these symptoms only after they have become frequent, persistent, and clinically troublesome, thereby contributing substantially to diagnostic delay [3].

These epidemiological data underscore the clinical consequences of delayed diagnosis. The five-year relative survival rate exceeds 90–93% when ovarian cancer is detected at stage I, while it decreases to approximately 20% once the disease is diagnosed at an advanced stage. Despite the major prognostic benefit associated with early intervention, only about 25% of cases are currently identified at the earliest, most treatable stage [4,5].

Ovarian cancer is not a single histological entity but rather a heterogeneous group of malignancies. Epithelial ovarian cancer accounts for approximately 90% of cases, yet this category includes several histotypes with distinct molecular characteristics, metastatic behaviors, and putative cells of origin, including high-grade serous ovarian carcinoma (HGSOC), endometrioid carcinoma, clear cell carcinoma, mucinous carcinoma, and low-grade serous carcinoma [6].

This histological and biological heterogeneity complicates both biomarker development and the implementation of standardized diagnostic assays. A molecular signal that performs well in HGSOC cannot automatically be assumed to show comparable performance in non-serous histologies, in which tumor shedding, epigenetic landscapes, and circulating analyte abundance may differ substantially [7,8,9].

Liquid biopsy has developed rapidly in response to the unmet need for a minimally invasive and biologically informative diagnostic approach. By detecting tumor-derived analytes in blood and other body fluids, liquid biopsy may contribute to earlier diagnosis, more refined biological stratification, and more dynamic disease monitoring than conventional serological testing. In ovarian cancer, its evolving role should be considered across three related but distinct clinical contexts: population screening in asymptomatic women, preoperative triage of women presenting with adnexal masses, and post-treatment monitoring for molecular residual disease and early relapse [10,11].

### Review Approach and Literature Selection

This article is a narrative review informed by a structured literature search of PubMed, Scopus, and Web of Science. The search was updated through January 2026 using combinations of the terms ovarian cancer, epithelial ovarian cancer, liquid biopsy, circulating tumor DNA, cell-free DNA, methylation, early detection, screening, adnexal mass, triage, molecular residual disease, surveillance, multi-cancer early detection, and clonal hematopoiesis. Priority was given to ovarian-specific original studies, prospective cohorts, validation studies, guidelines, and registry-listed ongoing prospective trials. Broader pan-cancer or mixed gynecologic studies were retained only when they provided methodological or translational context directly relevant to ovarian cancer and were identified as such in the text. Because assay platforms, clinical settings, comparator populations, and endpoints were highly heterogeneous, the evidence was synthesized narratively rather than meta-analytically.

## 2. The Limitations of Traditional Diagnostics: CA-125, Transvaginal Ultrasound, and Triage Algorithms

The evaluation of serum CA-125, a high-molecular-weight transmembrane glycoprotein, has long been the mainstay of non-invasive ovarian cancer diagnosis. Early assessments showed that 82% of patients with surgically proven ovarian cancer had increased serum CA-125 levels, compared to only 1% of healthy individuals and 6% of patients with benign tumors. The assay was first created as a diagnostic tool in 1981 by Bast and colleagues. Although CA-125 is still widely used in modern clinical practice, major shortcomings in both sensitivity and specificity severely limit its usefulness as an early diagnostic or population screening tool [12].

CA-125 has strong sensitivity in cases of advanced-stage illness (Stages III and IV), with increased levels seen in 84.2% to 92% of patients. Because of this, the biomarker is very useful for tracking chemotherapeutic response over time and identifying macroscopic disease recurrence. However, the diagnostic sensitivity of CA-125 falls to 50% to 62% in early-stage illness (Stages I and II). This indicates that the test is essentially ineffective for early interception because over half of all patients with extremely treatable, localized ovarian cancer have absolutely normal CA-125 levels [13,14,15].

Moreover, CA-125 has limited clinical specificity. A wide range of benign physiological states and inflammatory conditions, including menstruation, endometriosis, pelvic inflammatory disease, diverticulitis, and benign adnexal cysts, can increase serum levels. Interpretation is further complicated by menopausal status: the marker generally performs better in postmenopausal populations, whereas false-positive elevations are substantially more common in premenopausal women, making universal thresholds problematic [16,17,18].

Prominent international guidelines, including those from the National Comprehensive Cancer Network (NCCN) and the European Society for Medical Oncology (ESMO), do not support the use of CA-125 or transvaginal ultrasound for routine population screening in asymptomatic women because these approaches have not demonstrated a mortality benefit and can generate substantial harm from false-positive results [19].

Rather, current guidelines reserve CA-125 and ultrasound primarily for the evaluation of symptomatic patients, adnexal-mass triage, and disease monitoring rather than routine population screening [19]. These settings should not be conflated, because population screening in asymptomatic women, diagnostic triage in women with a pelvic or adnexal mass, and post-treatment surveillance each require different performance thresholds, comparators, and clinical end points. In parallel, contemporary management of epithelial ovarian cancer increasingly incorporates molecular characterization—including BRCA1/2 status and other predictive biomarkers where clinically indicated—because recurrent disease can evolve biologically and therapeutic stratification cannot be based on CA-125 alone [19,20]. Clinicians therefore use multivariate algorithmic models to address the diagnostic limitations of standalone CA-125 testing at initial presentation. These models are intended to stratify malignancy risk in patients with indeterminate pelvic masses and to support referral decisions regarding specialized gynecologic-oncology care [21,22,23,24].

Diagnostic accuracy can be improved by incorporating additional protein biomarkers, particularly human epididymis protein 4 (HE4). In women with adnexal masses, HE4 is generally more specific than CA-125 and may be less frequently elevated in benign conditions such as endometriosis, although performance still varies by clinical setting and disease stage [25]. Because endometriosis may coexist with, or represent the background lesion for, a subset of ovarian neoplasms, biomarker interpretation in endometriosis-associated adnexal masses should remain integrated with clinicopathologic assessment rather than being reduced to a single serum marker [26]. The principal triage algorithms currently used in women with adnexal masses differ in their required clinical inputs, intended diagnostic function, and overall balance between sensitivity and specificity, as summarized in Table 1.

As reflected in Table 1, these algorithms improve preoperative risk stratification but do not eliminate clinically relevant misclassification. Available data indicate that more than 40% of patients referred to specialized oncologic surgical centers because of high-risk indices are ultimately found to have benign disease at final histology. This false-positive referral burden carries psychological consequences for patients, can trigger repeat imaging or invasive work-up, and generates avoidable pressure on specialist healthcare systems [25].

## 3. The Biological Mechanisms and Complex Dynamics of Circulating Tumor DNA Shedding

The basic concept of liquid biopsy technology is the identification, separation, and sequencing of components linked to tumors that are either actively or passively secreted into physiological fluids. Although this includes circulating tumor cells (CTCs), cell-free RNA, exosomes, and tumor-educated platelets (TEPs), cell-free DNA (cfDNA) is the most thoroughly studied and clinically promising analyte. Background cfDNA is constantly released into the peripheral circulation in healthy persons, mostly as a result of hematopoietic cells undergoing normal apoptosis. A small portion of this total cell-free nucleic acid pool in cancer patients comes directly from the main tumor, distant disseminated tumor cells, or regional lymph node metastases; this particular malignant fraction is known as circulating tumor DNA (ctDNA) [27]. The intrinsic tumor biology, the surrounding tumor microenvironment, and systemic anatomical barriers interact in a very complicated way to control the active shedding of ctDNA into the bloodstream. The basic characteristics of high-grade serous ovarian cancer include large regions of hypoxia-induced necrosis, high mitotic indices, and fast, unchecked cellular growth [28]. Disproportionately large amounts of broken DNA and cellular debris are often released into the extracellular space by tumors with such fast cellular turnover and significant microscopic necrosis. As a result, ovarian cancer exhibits a comparatively high overall ctDNA detection rate among different solid tumors, ranging from roughly 75% to 90% in the advanced, metastatic setting [29].

However, there are particular physiological obstacles to the effectiveness of liquid biopsy due to the anatomical placement of early-stage ovarian cancer. The direct, highly vascularized access to the systemic circulation that is typical of cancers originating in the liver, lungs, or gastrointestinal tract is frequently absent from tumors that are entirely limited to the peritoneal cavity. Additionally, there are strong biological and physical barriers in the local tumor microenvironment that prevent DNA from entering the peripheral bloodstream [30]. Tumor DNA and apoptotic bodies can be captured, cleared, or enzymatically broken down locally by dense, fibrotic stromal tissue, vast extracellular mucin networks, and the very effective phagocytic activity of active immunological macrophages. Due to these obstacles, the mutant allele fraction (MAF) of ctDNA in highly localized Stage I disease may be well below 0.1% of the total cfDNA pool. In ultra-sensitive tumor-guided assays for epithelial ovarian cancer, ctDNA fractions down to 0.0004% have been trackable, underscoring the technical difficulty of early-stage detection [31].

Clonal hematopoiesis of indeterminate potential (CHIP) is a major systemic confounder in ctDNA analysis. Blood cells, particularly in older individuals, can acquire somatic mutations in genes such as TP53, DNMT3A, TET2, ASXL1, and PPM1D. If plasma cfDNA sequencing is not compared with matched leukocyte DNA, these hematopoietic variants may be misclassified as tumor-derived ctDNA, leading to false-positive results and misleading biological interpretations [32]. This issue is particularly relevant in ovarian cancer because TP53 is both the dominant driver of high-grade serous carcinoma and a recognized CHIP-associated gene. In practice, reliable mitigation requires parallel sequencing of matched leukocyte DNA, rigorous background error suppression, and cautious interpretation of very-low-allele-fraction plasma-only variants [32,33].

## 4. Genomic Alterations and Alternative Fluid Sampling: The TP53 Paradigm

Mutations in *TP53* dominate the genetic landscape of high-grade serous ovarian cancer and are present in almost all HGSOCs (~96%) [34]. In matched analyses, plasma–tumor mutational concordance has been reported at 83% at diagnosis and 90% at relapse, supporting the use of ctDNA as a molecular proxy of the underlying tumor genome [35].

**Figure 1:**
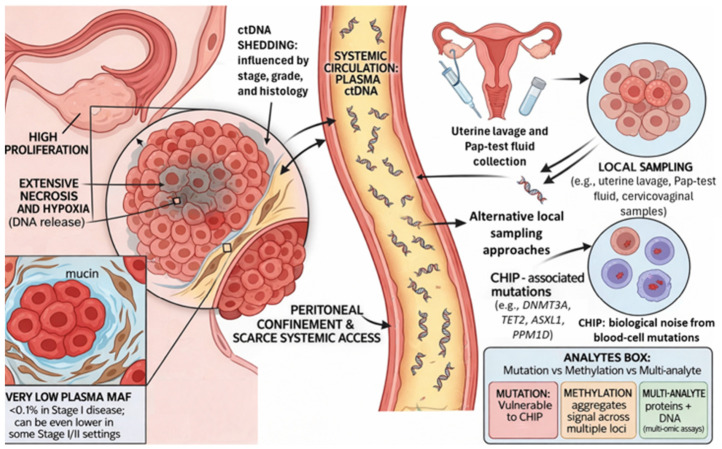
Biological and technical determinants of ctDNA detectability in ovarian cancer and rationale for alternative local sampling strategies. Schematic overview of the main factors influencing ctDNA analysis in ovarian cancer. On one hand, aggressive tumor features such as high proliferation, extensive necrosis, and hypoxia may increase DNA release. On the other hand, in early-stage disease, anatomical confinement to the peritoneal cavity, stromal and microenvironmental barriers, local DNA degradation, and phagocytic clearance may substantially limit ctDNA entry into the peripheral circulation, resulting in very low plasma mutant allele fractions. The figure also highlights the potential diagnostic rationale for alternative local sampling approaches, including uterine lavage, Pap test fluid, and cervicovaginal sampling, which may increase tumor-proximal molecular signal capture. Finally, clonal hematopoiesis of indeterminate potential (CHIP) is shown as a major source of biological background noise that can confound plasma-based mutation analysis if matched leukocyte controls are not included. Abbreviations: cfDNA, cell-free DNA; CHIP, clonal hematopoiesis of indeterminate potential; ctDNA, circulating tumor DNA; MAF, mutant allele fraction.

The dynamic evolutionary patterns of *TP53* variants detected in ctDNA can map the genetic trajectories of relapse-seeding clones, distinguish true progression from transient radiological ambiguity, and reveal the distribution of subclonal populations across metastatic sites [36]. As illustrated in Figure 1, these limitations of plasma-based ctDNA detection provide the biological rationale for exploring tumor-proximal sampling strategies, including uterine, endocervical, and cervicovaginal approaches.

In a mixed cohort study of women with endometrial or ovarian malignancies, PapSEEK used multiplexed mutation testing and aneuploidy analysis on cervical or intrauterine samples rather than a conventional blood-based ctDNA workflow [37]. Importantly, the often-cited diagnostic figures should not be interpreted as direct estimates of ovarian-cancer screening performance in an unselected screening population. Within the ovarian-cancer subset, sensitivity was 33% with endocervical Pap-brush samples and 45% with intrauterine Tao-brush samples, while specificity remained approximately 99–100% [37]. These data are best interpreted as proof-of-concept for tumor-proximal sampling, because the study included mixed gynecologic cohorts, the ovarian subsets were smaller than the endometrial cohorts, and performance varied according to sampling method. Consistent with this, reviews of cervicovaginal-fluid biomarker studies have highlighted technical feasibility but also emphasized the continuing need for ovarian-specific validation, stage-stratified reporting, and sampling standardization before these approaches can be positioned clinically [38].

## 5. Epigenetic Signatures: Harnessing DNA Methylation as an Early Event

Because tumor-derived mutations can be extremely sparse in early-stage disease, substantial attention has shifted toward epigenetic alterations, especially DNA methylation. Aberrant methylation is an early and recurrent feature of ovarian tumorigenesis, and promoter methylation of tumor-suppressor genes such as OPCML has been repeatedly explored as a biomarker candidate in ovarian cancer [39,40].

Compared with genomics, epigenetic profiling may offer a practical diagnostic advantage because methylation signals can be integrated across multiple loci, allowing mathematically aggregated classifiers to remain informative even when individual regions contribute only modest signal [39,40,41,42].

Compared with single-variant mutation assays, methylation-based approaches can interrogate multiple CpG sites within the same differentially methylated region, which may increase analytical signal in low-shedding settings. This feature helps explain the growing interest in cfDNA methylation assays for early detection, although clinical performance still requires careful prospective validation [39,41,42].

The potential diagnostic advantage of methylation analysis over conventional serology has been demonstrated in clinical evaluations. In one head-to-head study using the ELSA-seq platform to analyze targeted differentially methylated regions (DMRs), a cfDNA methylation model achieved an overall sensitivity of 94.7%, a specificity of 88.7%, and an area under the receiver operating characteristic curve (AUC) of 0.967, outperforming both CA-125 alone (AUC 0.863) and the ROMA (AUC 0.900) within the same cohort [41,42].

As summarized in Table 2, currently available blood-based approaches should not be interpreted as directly comparable diagnostic tests, because the reported metrics derive from different cohort compositions, clinical settings, assay platforms, thresholds, and intended uses. Most ovarian-cancer datasets remain case–control, hospital-based, or symptom-enriched, meaning that sensitivity, specificity, and AUC cannot be translated directly into screening positive predictive value. This distinction is especially important for stage I disease, where performance remains the central clinical bottleneck and the evidence is usually weakest. Conventional serum markers such as CA-125 remain clinically relevant in symptomatic women and in adnexal-mass evaluation, but their limited sensitivity in stage I disease and reduced specificity in benign gynecologic or inflammatory conditions prevent their use as stand-alone screening tools. By contrast, mutation-based ctDNA assays offer high biological specificity when true tumor-derived variants are detected, yet their performance in early-stage ovarian cancer remains constrained by low tumor shedding and very low allele fractions [33,43,44,45]. In addition, these assays are vulnerable to CHIP-related background noise, which can further limit their value as isolated diagnostic tests [32,33].

These limitations help explain the growing interest in cfDNA methylation models and broader multi-analyte strategies, which may capture diagnostically informative signals even when single mutations are too sparse to support reliable early detection. In ovarian cancer, the most plausible near-term role of liquid biopsy is therefore not as a universal stand-alone screening assay, but as an integrated component of clinically defined pathways, particularly adnexal-mass triage, molecular risk stratification, and, potentially, post-treatment surveillance. Ovarian-focused methylation assays and multi-omic models such as EarlySEEK and AKRIVIS GD™ appear especially relevant in this regard, whereas pan-cancer platforms such as CancerSEEK/DETECT-A and Galleri should currently be viewed primarily as proof of principle for high-specificity blood-based detection rather than as validated ovarian-specific screening strategies [41,42,45,46,47,48,49,50,51,52,53,54,55,56].

## 6. From Single-Analyte Assays to Multi-Omic Liquid Biopsy Strategies

**Figure 2:**
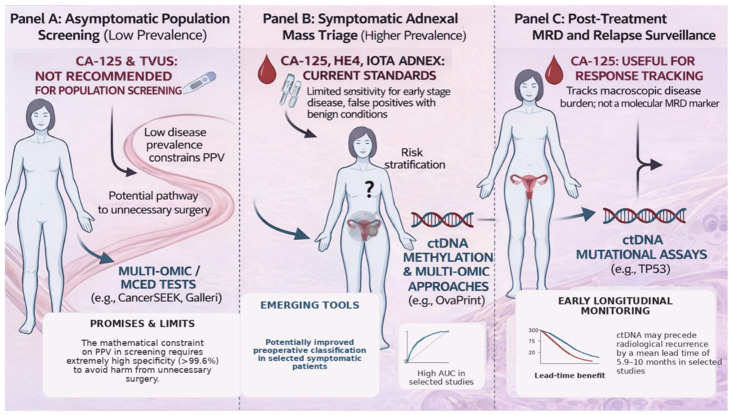
Clinical settings for liquid-biopsy application in ovarian cancer: screening, adnexal-mass triage, and post-treatment molecular surveillance. Schematic overview of the three main clinical contexts in which liquid-biopsy approaches may be applied in ovarian cancer. Panel A summarizes the population-screening setting in asymptomatic women, where low disease prevalence imposes stringent specificity and positive predictive value requirements, and where conventional tools such as CA-125 and transvaginal ultrasound are not recommended for routine stand-alone screening. Panel B illustrates the symptomatic adnexal-mass setting, in which liquid-biopsy approaches based on cfDNA methylation, multi-omic models, and machine-learning integration may have greater short-term clinical relevance as adjuncts to preoperative triage alongside established algorithms such as ROMA and IOTA ADNEX. Panel C summarizes the post-treatment setting, where ctDNA mutation-based assays may enable molecular residual disease assessment and earlier detection of recurrence compared with conventional biomarkers such as CA-125, although prospective interventional validation remains necessary. Abbreviations: AUC, area under the curve; CA-125, cancer antigen 125; cfDNA, cell-free DNA; ctDNA, circulating tumor DNA; IOTA, International Ovarian Tumor Analysis; MRD, molecular residual disease; PPV, positive predictive value; ROMA, Risk of Ovarian Malignancy Algorithm; TVUS, transvaginal ultrasound.

Liquid-biopsy development has progressively shifted from single-analyte assays toward multi-omic approaches that combine genomic, epigenetic, proteomic, or lipidomic signals within the same diagnostic framework. The rationale for this transition is that integrated models may partially compensate for the limitations of individual analytes and improve performance in clinically complex settings such as early detection and adnexal-mass triage [49]. The major clinical settings in which liquid-biopsy strategies may be applied in ovarian cancer, together with their principal opportunities and limitations, are summarized schematically in Figure 2.

### 6.1. The EarlySEEK Multi-Analyte Approach

The EarlySEEK assay provides a useful example of why numerical harmonization is important in this field. In the original study, sensitivities of 58.7% and 79.0% at 95% specificity referred to ctDNA alone and CA125 alone, respectively, whereas 85.5% referred to the intermediate two-analyte CA125 + ctDNA model [45]. By contrast, the commonly cited 94.2% sensitivity at 95.1% specificity and AUC 0.945 referred to the full EarlySEEK classifier, which added HE4, CA19-9, prolactin, and IL-6 to the CA125 + ctDNA backbone and outperformed ROMA (AUC 0.853) in the same symptomatic cohort [45]. These figures therefore describe related but distinct models rather than discrepant results. Even so, the study population consisted of women with pelvic masses in an enriched diagnostic setting, so the findings are most relevant to preoperative triage rather than to general-population screening.

### 6.2. CancerSEEK and the DETECT-A Study

This multi-marker concept is extended beyond gynecological malignancies by the CancerSEEK platform. CancerSEEK evaluates 16 cancer-associated genes, including major drivers such as *TP53* and *KRAS*, together with eight protein markers (CA-125, CEA, CA19-9, prolactin, HGF, osteopontin, myeloperoxidase, and TIMP-1) using multiplex PCR plus immunoassay-based protein measurement. A random-forest classifier is then applied to the integrated data matrix to generate both a cancer-probability score and a tissue-of-origin prediction [48,50].

Although CancerSEEK showed only modest pan-cancer sensitivity in stage I disease, the original case–control study reported a 98% sensitivity for ovarian cancer, supporting the potential of multi-analyte designs while still requiring cautious interpretation in relation to real-world screening settings. The prospective DETECT-A study, a large interventional trial that enrolled 10,006 asymptomatic women aged 65 to 75 years, evaluated the feasibility and diagnostic performance of integrating a multi-cancer blood test with confirmatory PET/CT in a real-world screening workflow [50]. The assay maintained a strong specificity of 98.9% and a negative predictive value (NPV) of 99.3% when using the blood test alone. After PET/CT imaging was used to assess positive molecular signals, the final diagnostic specificity increased to 99.6% and the positive predictive value (PPV) doubled from 19.4% to 28.3%. Within this diagnostic pathway, 65% of detected malignancies were identified at stage I/II; however, the study was designed to assess feasibility and downstream diagnostic consequences rather than mortality benefit, and its multi-cancer results should not be conflated with ovarian-specific triage data [50].

### 6.3. AKRIVIS GD™ and Multi-Omic Ganglioside Biomarkers

AOA Dx has developed AKRIVIS GD™, a proprietary multi-omic blood-based assay built on the GlycoLocate™ platform for the evaluation of symptomatic women with suspected ovarian cancer. The assay integrates lipidomic and proteomic features, building on prior evidence that tumor-associated gangliosides such as GD2 and GD3 may represent diagnostically informative biomarkers in epithelial ovarian cancer [51]. In a peer-reviewed clinical evaluation of 509 serum samples from a clinically relevant symptomatic cohort, the model achieved a sensitivity of 98.7% for early-stage ovarian cancer and 98.6% across stages and histologic subtypes at a fixed specificity of 70%, suggesting potential value within triage-oriented diagnostic pathways at first clinical presentation in women with nonspecific but concerning symptoms [52]. However, the fixed specificity of 70% is far below what would be required for population screening, so the assay should presently be framed as a possible adjunct for symptomatic triage rather than as a screening platform.

## 7. Multi-Cancer Early Detection (MCED): The Galleri Test and CCGA/PATHFINDER Trials

The Galleri test, developed by GRAIL, is one of the best characterized multi-cancer early detection (MCED) platforms and is based on analysis of cfDNA methylation patterns rather than targeted mutation panels. It was developed within the Circulating Cell-free Genome Atlas (CCGA), a large prospective case–control program that enrolled approximately 15,000 participants across the United States and Canada, and uses machine-learning algorithms not only to classify samples as cancer signal detected or not detected, but also to predict the cancer signal origin (CSO), thereby helping to guide the subsequent diagnostic work-up [53,54,55,56].

In CCGA substudy 1, multiple cfDNA analytical strategies were compared head-to-head, and whole-genome methylation showed the strongest performance among the evaluated cfDNA analytical strategies at 98% specificity, providing the basis for development of the final targeted methylation assay used in later validation studies. In the independent CCGA validation set, this targeted methylation-based test achieved 99.5% specificity, 51.5% overall sensitivity, and stage-dependent sensitivities of 16.8%, 40.4%, 77.0%, and 90.1% for stages I, II, III, and IV, respectively; importantly, cancer signals were detected across more than 50 tumor types and cancer-signal origin prediction was correct in 88.7% of true-positive cases [53,54,55].

From an ovarian-cancer perspective, these platforms are most relevant as proof that methylation-based cfDNA assays can sustain very high specificity in early-detection workflows. However, ovarian-specific performance estimates are less mature than the headline pan-cancer metrics reported in CCGA and PATHFINDER, so extrapolation to ovarian-cancer screening should remain cautious until disease-specific prospective evidence becomes available [53,54,55,56].

Prospective implementation data from PATHFINDER further support the feasibility of integrating this assay into real-world screening workflows. In that study, conducted in 6621 participants with analyzable results, a cancer signal was detected in 92 individuals; the overall specificity was 99.1%, the negative predictive value was 98.6%, and the positive predictive value was 38.0%, while the refined test version improved specificity to 99.5% and yielded a PPV of 43.1%. Notably, the study was designed primarily to assess feasibility and downstream diagnostic consequences rather than mortality benefit, and therefore its results are best interpreted as evidence that MCED testing may complement, rather than replace, established single-cancer screening strategies [53,54,55,56].

### 7.1. Artificial Intelligence (AI) Integration

Machine-learning approaches are increasingly used in contemporary multi-omic cancer diagnostics because the simultaneous integration of large methylation datasets, high-dimensional small RNA sequencing outputs, and dynamic proteomic or lipidomic measurements exceeds the practical limits of conventional single-variable or low-dimensional statistical frameworks. In this context, machine-learning approaches are increasingly incorporated into assay development rather than being used only as secondary statistical tools. A clear example is provided by transformer-based models applied to cfDNA methylation profiling: in a recent ovarian cancer study, the pretrained MethylBERT architecture learned genome-wide methylation structure from more than 110,000 whole-genome and reduced-representation bisulfite sequencing datasets and was then fine-tuned for early epithelial ovarian cancer detection, suggesting that deep-learning models may capture diagnostically relevant patterns not easily identified by conventional feature-selection strategies alone. Similarly, neural network models trained on serum miRNA-seq data have achieved strong diagnostic performance in ovarian cancer, with reported AUC values of 0.90 in the main sequencing cohort and approximately 0.92–0.93 in comparative analyses, while performing independently of CA-125. In parallel, sequencing-based methylation classifiers such as OvaPrint have shown high rule-in performance in women with adnexal masses, achieving a positive predictive value of 95% for high-grade serous ovarian cancer, supporting the idea that machine-learning-based molecular classification could contribute meaningfully to next-generation diagnostic development rather than simply refining conventional biomarker analysis [47,57,58,59].

### 7.2. Statistical Realities: The Positive Predictive Value in Screening

Although several contemporary liquid-biopsy assays show high reported sensitivity and specificity, their real clinical value, and ultimately their suitability for implementation, depends on the clinical setting in which they are used. In ovarian cancer, it is essential to distinguish population screening in asymptomatic women, diagnostic triage in women who already present with a symptomatic adnexal mass or suspicious imaging findings, and post-treatment monitoring after established disease. These use cases have fundamentally different prevalence structures, harms of false positivity, and evidentiary standards. In the triage setting, the pretest probability of malignancy is substantially higher because the tested population is already enriched for clinically suspicious cases; under these conditions, sensitivity, specificity, and AUC remain highly informative and clinically useful for directing referral and surgical planning. By contrast, when the same assay is applied to population screening, performance must be interpreted against the very low background prevalence of ovarian cancer in asymptomatic postmenopausal women, historically estimated at approximately 1 in 2500 [60].

This distinction has major mathematical consequences. By Bayes’ theorem, PPV is tightly constrained by disease prevalence, such that even a highly accurate test may generate a substantial number of false-positive results when deployed in a low-prevalence population. Indeed, with a prevalence of roughly 1:2500, a screening strategy must achieve a PPV of at least 10% to remain clinically acceptable, which in practice requires specificity of about 99.6% even under highly favorable assumptions about sensitivity; some reviews round this requirement to approximately 99.7%. Put differently, a PPV of 10% means that for every true ovarian cancer detected, about nine women without ovarian cancer would nonetheless test positive and could therefore be exposed to unnecessary repeat testing, imaging, invasive procedures, or surgery. For this reason, assays that appear highly promising in enriched symptomatic cohorts cannot be assumed to be suitable for general-population screening without separate validation in true screening populations and careful evaluation of their predictive values, downstream harms, and clinical utility [61,62].

A false-positive result can have important clinical consequences in ovarian cancer screening, because it may trigger repeat imaging, specialist referral, invasive diagnostic procedures, and, in a substantial proportion of women, unnecessary surgery with nontrivial morbidity. In the PLCO trial, false-positive screening results led many women to operative work-up, and major complications occurred in 15.1% of surgeries performed after false-positive tests; the USPSTF likewise concluded that the harms of ovarian cancer screening are at least moderate and may be substantial, largely because false-positive results can lead to unnecessary surgical intervention in women without cancer [63,64].

This issue is particularly important because predictive values can be seriously distorted when test performance is inferred from datasets that do not reflect the prevalence of disease in the intended-use population. A well-known example is OvaSure, for which subsequent methodological critiques showed that the originally claimed positive predictive value was incompatible with real-world screening prevalence and effectively corresponded to a disease prevalence close to 50%; after reappraisal, the estimated performance fell to 84% sensitivity, 95% specificity, and a PPV of only 6.5%, and the test was withdrawn [65,66]. This statistical constraint also explains why simple threshold adjustment of conventional biomarkers is insufficient to solve the screening problem. For CA-125, increasing the cutoff from 35 U/mL to 70 U/mL can raise specificity to about 99%, but this gain is achieved at the expense of sensitivity, which in one analysis fell from 75% to 70%, underscoring the unavoidable trade-off between reducing false positives and missing clinically important cancers [15,18]. For this reason, the appeal of more complex multi-omic and MCED platforms lies not simply in higher analytical sophistication, but in their ability to sustain extremely high specificity while preserving useful sensitivity. In PATHFINDER, the refined Galleri assay achieved a specificity of 99.5% and a positive predictive value of 43.1%, supporting a substantially higher level of confidence in a positive result than would be possible with conventional single-marker approaches alone, although these findings should still be interpreted within the context of a feasibility study rather than as definitive proof of screening benefit [56]. Restricting testing to women at substantially elevated baseline risk, such as BRCA1/2 carriers, would also increase pretest probability and therefore improve positive predictive value in Bayesian terms; however, this should not be interpreted as evidence that ovarian cancer screening is already effective in that population, because available studies and guidelines continue to conclude that screening has not been shown to reduce mortality even in high-risk BRCA1/2 carriers [19,67].

## 8. Health Economics, Cost-Effectiveness, and the Financial Burden of Ovarian Cancer

The integration of advanced liquid-biopsy platforms into clinical guidelines will depend not only on analytical performance and clinical validity but also on whether they deliver acceptable value at the health-system level. In ovarian cancer, this question is especially important because the economic burden is concentrated at the beginning and end of the disease trajectory. In a recent U.S. phase-of-care analysis, mean costs exceeded US$ 200,000 per patient-year during the initial care phase and remained above US$ 129,000 during end-of-life care. Beyond direct medical expenditure, a 2025 multinational analysis estimated approximately US$ 70 billion in socioeconomic losses attributable to ovarian cancer across 11 countries, with more than 90% of that burden driven by premature mortality. Together, these data explain why stage shift, rather than analytical novelty alone, is the central economic promise of early detection [68,69]. From a health economic perspective, the key question is not whether a test is technologically sophisticated, but whether it can move diagnosis toward earlier, more treatable disease at a cost compatible with accepted willingness-to-pay thresholds. In a U.S. modeling study informed by UKCTOCS, multimodal ovarian-cancer screening associated with a 15% mortality reduction yielded incremental cost-effectiveness ratios between US$ 106,187 and US$ 155,256 per quality-adjusted life year (QALY) gained. In the original UK economic analysis, cost-effectiveness remained highly sensitive to assumptions regarding screening cost, long-term mortality benefit, and extrapolation beyond trial follow-up. More recent U.S. MCED modeling likewise suggested that sequencing-based multi-cancer testing could be cost-effective under favorable assumptions, with one analysis estimating an ICER of 66,048 US$/QALY at a list price of 949 US$. However, these projections were strongly dependent on the magnitude of stage shift, survival benefit, and future cancer-management costs [70,71,72]. Recent systematic-review evidence also confirms that ovarian-cancer screening can generate false-positive results leading to unnecessary surgery, a factor that must be incorporated into any serious economic appraisal [73]. The Kentucky ovarian screening program illustrates this point. The base cost per screen was estimated at US$ 56 and increased to US$ 65.27 after accounting for surgery in false-positive cases. It fell to approximately US$ 40.96 locally and US$ 48.58 in a nationalized estimate only after incorporation of the projected economic benefits of earlier-stage treatment, regained earnings, and recovered tax contributions. Although these data are encouraging, they derive from a long-running ultrasound-based screening program and should not be extrapolated uncritically to more complex multi-omic assays [74]. Overall, the economic case for advanced liquid-biopsy platforms will depend on prospective evidence showing that earlier detection produces meaningful stage shift, an acceptable downstream diagnostic burden, and payer-credible cost-effectiveness, rather than on analytical novelty alone [70,71,72,73,74].

## 9. Regulatory Frameworks, Standardization, and the BLOODPAC Initiative

The clinical translation of complex liquid-biopsy assays is increasingly constrained not only by analytical sensitivity, but also by the absence of universal standardization across the pre-analytical and analytical workflow. Blood collection tube type, the choice of plasma versus serum, time to plasma separation, centrifugation schemes, storage and transport conditions, freeze–thaw cycles, DNA extraction methods, library preparation chemistry, sequencing depth, and downstream bioinformatic processing can all materially influence cfDNA yield, background noise, and variant detection. This is particularly relevant in ctDNA analysis, where the tumor-derived fraction may be extremely low: under low-allele-fraction conditions, even modest contamination by leukocyte-derived genomic DNA can dilute the effective variant allele fraction and reduce the reproducibility of rare-event detection. Likewise, inadequate error suppression or insufficient molecular barcoding may inflate false-positive calls when mutant allele fractions approach or fall below 0.1%. Accordingly, the need for tightly controlled pre-analytical workflows including appropriate tube selection, rapid or stabilizing sample handling, double-spin plasma processing where appropriate, and standardized downstream analysis has become a central requirement for robust clinical implementation rather than a purely technical consideration [46,75,76,77,78,79,80,81,82].

## 10. Prospective Studies and Emerging Clinical Pathways in Ovarian Cancer Liquid Biopsy

Prospective evidence is beginning to clarify the most plausible clinical roles of liquid biopsy in ovarian cancer, although the field is still dominated by prospective longitudinal and hospital-based diagnostic studies rather than by randomized interventional trials. In the preoperative setting, a prospective hospital-based diagnostic study of tumor-educated platelets (TEPs) enrolled 761 treatment-naïve inpatients with adnexal masses across nine centers and showed that platelet RNA profiles could discriminate ovarian cancer with high accuracy, with an AUC of 0.918 in the combined validation cohort and 0.922 when combined with CA125. These findings support the concept that blood-based assays may add objective molecular information to conventional adnexal-mass triage, rather than simply replacing ultrasound-based assessment. More broadly, multimodal strategies integrating blood-based biomarkers, including ctDNA features, with radiomics and clinical variables have also shown feasibility in high-grade serous ovarian cancer, suggesting that future diagnostic pathways may rely on complementary molecular and imaging data streams rather than on any single biomarker class alone [83,84]. An equally important area of development is the post-treatment setting, where ctDNA is increasingly being investigated as a marker of molecular residual disease and early recurrence. In a prospective study of TP53-mutated ctDNA in high-grade serous ovarian carcinoma, serial plasma monitoring supported the utility of ctDNA as a non-invasive biomarker of treatment response. Likewise, a prospective longitudinal ctDNA workflow applied to 78 plasma samples from 12 patients demonstrated that ctDNA profiling could reveal clinically actionable alterations during treatment, with potentially targetable events identified in 58% of patients; notably, in one chemoresistant case, detection of ERBB2 amplification led to treatment modification followed by tumor shrinkage and normalization of CA125. Together, these studies provide proof of concept that longitudinal ctDNA analysis may contribute not only to disease monitoring, but also to dynamic therapeutic stratification in selected patients [85,86]. The strongest near-term clinical application may, however, lie in relapse prediction after completion of primary therapy. In one longitudinal surveillance study, ctDNA was detected only in patients who eventually relapsed and preceded radiological recurrence by an average of 10 months; post-surgical ctDNA positivity was strongly associated with shorter recurrence-free survival, whereas CA125 was not significantly predictive in the same cohorts. More recently, tumor-guided plasma DNA analysis in 63 patients with epithelial ovarian cancer showed that positive ctDNA in the last on-treatment sample was associated with more rapid progression and worse overall survival, and in a subset of patients ctDNA detected progression earlier than standard surveillance with a median lead time of 5.9 months. Taken together, these prospective data support a model in which liquid biopsy may contribute across the ovarian cancer continuum, from preoperative stratification to post-treatment molecular surveillance [87]. More broadly, this evolving paradigm is consistent with evidence from other epithelial malignancies, in which molecular analysis of liquid samples has also shown potential for recurrence-risk stratification during follow-up [88]. However, the key unmet need is no longer simply earlier molecular detection, but prospective demonstration that acting on these signals translates into clinically meaningful benefit.

Among the most relevant registry-listed ongoing studies, the OVI-DETECT trial (NCT04971421) is evaluating whether a diagnostic algorithm integrating circulating tumor DNA (ctDNA) and tumor-educated platelets (TEPs) can improve the preoperative discrimination between benign adnexal masses and ovarian malignancy when combined with established triage tools, including ultrasound-based models such as RMI and IOTA and serum biomarkers such as CA125 and HE4. In parallel, NCT05801276 is assessing plasma CDO1 and HOXA9 methylation as a blood-based approach for ovarian cancer detection and assay validation, consistent with prior evidence supporting methylated HOXA9 as a diagnostically informative marker of ovarian malignancy. For clarity and practical reference, these ongoing prospective studies are summarized in dedicated Table 3, which reports the registry identifier, intended clinical setting, assay platform or analyte, target population, primary endpoint, planned enrollment, and current registry-reported study status [76,77,78,79,80,81,82,83,84,85,86,87,88,89].

Taken together, these prospective and ongoing studies suggest that the most plausible near-term implementation of liquid biopsy in ovarian cancer lies in clinically defined pathways spanning preoperative triage and post-treatment molecular surveillance. However, routine follow-up still relies largely on imaging and serum biomarkers, and the key unmet need is no longer simply to show that ctDNA can detect disease earlier, but to demonstrate in prospective interventional settings that acting on ctDNA-informed results improves clinically meaningful outcomes [87,88,89,90,91,92].

On the basis of the currently available evidence, a liquid-biopsy assay should meet at least four conditions before routine clinical adoption in ovarian cancer: first, a clearly defined intended use, such as population screening, symptomatic triage, or post-treatment surveillance; second, performance thresholds appropriate to that setting, including extremely high specificity and acceptable positive predictive value for screening; third, analytically robust workflows with standardized pre-analytics, adequate depth and error suppression, and matched leukocyte controls to mitigate CHIP-related false positives; and fourth, prospective evidence that assay-informed management improves patient-centered or survival-related outcomes at an acceptable downstream diagnostic and economic cost [61,62,63,64,70,71,72,73,74,75,76,77,78,79,80,87,88,89,90,91,92].

## 11. Conclusions

The clinical management of ovarian cancer has long been constrained by reliance on biological and imaging signals that often emerge only after the disease has reached a clinically advanced stage. Conventional triage strategies based on CA-125 and ultrasound remain valuable in the evaluation of adnexal masses, but their performance in the earliest phases of disease is intrinsically limited by suboptimal sensitivity for stage I tumors and by reduced specificity in the presence of benign gynecologic or inflammatory confounders. ctDNA and other liquid-biopsy analytes offer a broader molecular framework for early detection and disease monitoring, yet their performance is shaped by fundamental constraints related to tumor shedding, histologic heterogeneity, clonal hematopoiesis, and assay standardization. As outlined in Figure 2, population screening, symptomatic triage, and post-treatment surveillance should not be treated as interchangeable use cases, because they differ substantially in biological rationale, performance requirements, and realistic prospects for near-term implementation. A further point that deserves emphasis is that ovarian-cancer biomarker development should remain anchored to clinicopathologic context. Experience across gynecologic pathology shows that precursor lesions, histotype-specific biology, and integrated morphomolecular classification materially influence risk interpretation; this is particularly relevant when considering endometriosis-associated ovarian neoplasia and broader precision-classification frameworks in gynecologic malignancies [26,93].

Likewise, the translation of molecular assays from analytical promise to clinical utility depends on how effectively they are embedded within defined diagnostic workflows. Although derived from non-ovarian settings, recent studies on molecular profiling of thyroid cytology and on methylation-based urinary assays illustrate a general translational principle: preoperative triage, threshold calibration, integration with cytology, and longitudinal validation are essential determinants of real-world actionability [94,95,96,97,98]. This caution is directly relevant to ovarian-cancer liquid biopsy, where assay performance must ultimately be judged not only by analytical discrimination, but by whether it improves clinically meaningful decisions.

From an ovarian-cancer-specific perspective, the next translational step is not simply to develop more analytically sensitive assays, but to validate how ctDNA mutation profiling, cfDNA methylation analysis, and multi-analyte models can be embedded into clearly defined clinical pathways, including adnexal-mass triage, post-treatment molecular residual disease assessment, and longitudinal relapse surveillance. Future studies should therefore prioritize ovarian-focused prospective cohorts, explicit stage-stratified reporting, standardized pre-analytical workflows, matched leukocyte controls to reduce CHIP-related false positives, and interventional designs capable of determining whether acting on molecular signals improves clinically meaningful outcomes beyond earlier molecular detection alone [99,100].

## Figures and Tables

**Table 1:** Established triage algorithms for adnexal masses: required inputs, intended clinical role, and major limitations.

Triage Algorithm	Required Clinical Inputs	Most Relevant Intended Clinical Role	Representative Diagnostic Profile *	Main Strengths	Main Limitations/Caveats
**Risk of Malignancy Index (RMI)** [21]	Serum CA-125, menopausal status, and selected TVUS features (e.g., multilocularity, solid areas, bilaterality, ascites, intra-abdominal metastases)	Initial risk stratification of adnexal masses and support for referral to gynecologic-oncology care	Long-established algorithm with generally good specificity, but lower sensitivity than newer multimarker or ultrasound-based models	Simple, familiar, inexpensive, and widely applicable	Performance depends on CA-125 and ultrasound interpretation; may underperform in early-stage disease and in indeterminate masses
**Risk of Malignancy Algorithm (ROMA)** [22]	Serum CA-125, serum HE4, and menopausal status	Biochemical triage of women with adnexal masses, especially when distinguishing malignancy from benign gynecologic disease	Reported overall sensitivity is approximately 86.2% in representative studies, but performance remains less robust in stage I disease	Improves on CA-125 alone; HE4 may be less frequently elevated in benign conditions such as endometriosis	Accuracy varies by population and menopausal status; not sufficient as a stand-alone screening strategy
**IOTA ADNEX model** [23]	Age, serum CA-125, type of center, and six ultrasound variables	Detailed preoperative classification of adnexal masses, including estimation of benignity versus different malignant subtypes	Provides probabilistic discrimination between benign, borderline, stage I, stage II–IV, and secondary metastatic tumors	More granular than purely biochemical models; particularly useful for structured preoperative decision-making	Highly dependent on ultrasound quality and operator expertise; broader implementation may be limited outside experienced centers
**Multivariate Index Assay (MIA/OVA1)** [24]	Panel of five serum proteins, including CA-125 and additional non-specific markers	High-sensitivity referral support tool designed to reduce the risk of missing occult malignancy before surgery	Favors high sensitivity at the expense of lower specificity	Useful as a “safety-net” strategy in women planned for surgery	Lower specificity may increase false-positive referrals and unnecessary specialist work-up

* Performance varies across studies according to cohort composition, menopausal status, ultrasound expertise, thresholds, and intended use; values and descriptors are therefore provided as representative rather than directly comparable head-to-head metrics.

**Table 2:** Selected blood-based approaches relevant to ovarian cancer detection: representative studies, intended clinical setting, and major limitations.

Approach	Most Relevant Intended Clinical Setting	Representative Reported Performance *	Main Strengths	Main Limitations/Caveats
**CA-125** [12,13,14,15,16,17,18]	Symptomatic patients, adnexal-mass evaluation, longitudinal monitoring	Stage I/II sensitivity generally 50.0–62.0%; Stage III/IV sensitivity 84.2–92.0%	Widely available; clinically familiar; useful in follow-up and in combination with imaging/algorithms	Limited specificity in benign gynecologic and inflammatory conditions; performance influenced by menopausal status; unsuitable as a stand-alone population-screening test
**ctDNA mutation assays** [33,43,44,45]	Ovarian-focused plasma genotyping; enriched diagnostic cohorts; biological characterization	In one symptomatic cohort, ctDNA alone: 58.7% sensitivity at 95% specificity	High biological specificity when true tumor-derived variants are identified; may capture molecular heterogeneity	Stage I performance is particularly limited and highly variable; strongly dependent on tumor shedding; vulnerable to CHIP-related false positives; requires very deep sequencing and matched leukocyte controls
**cfDNA methylation classifiers** [41,42,46,47]	Ovarian-focused early detection and adnexal-mass triage	In a representative ELSA-seq study, 94.7% sensitivity, 88.7% specificity, AUC 0.967; other ovarian-focused methylation models remain promising but heterogeneous	Potentially stronger signal than single-mutation assays in low-shedding disease; multi-locus integration may improve robustness	Heterogeneous platforms and thresholds; limited prospective validation; stage I performance still requires rigorous verification; pre-analytical handling and bioinformatic standardization remain critical
**Multi-analyte protein + ctDNA model (EarlySEEK)** [45]	Symptomatic women with pelvic/adnexal masses; enriched diagnostic cohort; preoperative triage rather than general-population screening	At 95% specificity: ctDNA alone 58.7%, CA125 alone 79.0%, CA125 + ctDNA 85.5%; full EarlySEEK model 94.2% sensitivity at 95.1% specificity; AUC 0.945 versus 0.853 for ROMA	Combines orthogonal biological signals; improves on single-marker approaches and on CA125 + ctDNA/ROMA within the same study	Evidence derives from a symptomatic enriched cohort; stage-stratified external validation and prospective clinical-utility data are still lacking
**Pan-cancer multi-analyte model (CancerSEEK/DETECT-A)** [48,49,50]	MCED proof-of-concept; not ovarian-specific	In the original case–control study, ovarian-cancer sensitivity reached 98% at >99% specificity; in DETECT-A, blood test alone showed 98.9% specificity and 19.4% PPV, improving to 99.6% specificity and 28.3% PPV after PET/CT triage	Demonstrates feasibility of integrating molecular testing with confirmatory imaging; supports the multi-analyte concept	Not ovarian-specific; case–control and prospective interventional data should not be conflated; no evidence of mortality benefit
**Multi-omic lipid/protein model (AKRIVIS GD™)** [51,52]	Symptomatic women with possible ovarian cancer; first-presentation triage	98.7% sensitivity in early-stage disease and 98.6% overall sensitivity at 70% fixed specificity	Strong early-stage signal in a symptomatic cohort; multi-omic design may complement conventional triage	Specificity is too low for population screening; evidence is still limited to early clinical validation; broader independent validation is needed
**Pan-cancer methylation MCED model (Galleri/CCGA/PATHFINDER)** [53,54,55,56]	MCED proof-of-principle; ovarian-relevant but not ovarian-specific	In CCGA validation, 99.5% specificity and 51.5% overall sensitivity; stage-dependent sensitivity 16.8% (I), 40.4% (II), 77.0% (III), 90.1% (IV)	Very high specificity; strong proof that methylation-based assays can operate in early-detection workflows	Ovarian-specific performance remains insufficiently defined; low stage I sensitivity overall; current evidence supports complementarity, not replacement of disease-specific pathways

* Performance values derive from different study designs, cohort compositions, intended-use settings, thresholds, and endpoints; they are provided as representative metrics and should not be interpreted as direct head-to-head comparisons. Predictive values from symptomatic or case–control cohorts should not be extrapolated directly to population screening.

**Table 3:** Selected ongoing prospective studies discussed in the text: study identifier, clinical setting, assay platform/analyte, target population, primary endpoint, planned enrollment, and registry-reported current status.

Study/Identifier	Clinical Setting	Assay Platform/Analyte	Target Population	Primary Endpoint	Planned Enrollment	Current Status
**OVI-DETECT (NCT04971421)** [89]	Preoperative triage of suspected early ovarian malignancy/adnexal masses	ctDNA structural alterations and tumor-educated platelet RNA integrated with existing triage tools (RMI, IOTA, CA125, HE4)	Women ≥ 18 years referred for surgery for an ovarian tumor; obvious advanced-stage disease excluded in the registry summary	Diagnostic accuracy of the developed algorithm	450	Recruiting (registry listing)
**ctDNA methylation for detecting ovarian cancer (NCT05801276)** [90,91]	Diagnostic evaluation/methylation-based detection and assay verification	Plasma methylation assay for CDO1 and HOXA9, with comparison against histopathology, ROMA, and Sanger sequencing	Women ≥ 18 years undergoing surgery for pelvic or adnexal masses; registry also notes additional comparator gynecologic/solid-tumor cases	Diagnostic sensitivity/specificity and assay accuracy verification	>1400	Recruiting (registry listing)

## Data Availability

No new data were created or analyzed in this study.
